# A New Food Ingredient Rich in Bioaccessible (Poly)Phenols (and Glucosinolates) Obtained from Stabilized Broccoli Stalks

**DOI:** 10.3390/foods11121734

**Published:** 2022-06-14

**Authors:** Antonio Costa-Pérez, Diego A. Moreno, Paula M. Periago, Cristina García-Viguera, Raúl Domínguez-Perles

**Affiliations:** 1 Phytochemistry and Healthy Food Lab (LabFAS), Department of Food Science and Technology, CEBAS-CSIC, University Campus of Espinardo-25, 30100 Murcia, Spain; acosta@cebas.csic.es (A.C.-P.); dmoreno@cebas.csic.es (D.A.M.); rdperles@cebas.csic.es (R.D.-P.); 2 Associated Unit of Food Quality and Risk Assessment CEBAS-CSIC/UPCT, 30100 Murcia, Spain; paula.periago@upct.es; 3 Agronomic Engineering Department, Universidad Politécnica de Cartagena (UPCT), Paseo Alfonso XIII, 48, 30203 Cartagena, Spain

**Keywords:** broccoli stalks, processing, phytochemical fingerprinting, hydroxycinnamates, glucosinolates, bioaccessibility, UHPLC-ESI-QqQ-MS/MS, HPLC-DAD-ESI-MSn

## Abstract

Broccoli (*Brassica oleracea* var. *italica*) stalks account for up to 35% of the broccoli harvest remains with the concomitant generation of unused waste that needs recovery to contribute to the sustainability of the system. However, due to its phytochemical composition, rich in bioactive (poly)phenols and glucosinolates, as well as other nutrients, the development of valorization alternatives as a source of functional ingredients must be considered. In this situation, the present work aims to develop/obtain a new ingredient rich in bioactive compounds from broccoli, stabilizing them and reducing their degradation to further guarantee a high bioaccessibility, which has also been studied. The phytochemical profile of lyophilized and thermally treated (low-temperature and descending gradient temperature treatments), together with the digested materials (simulated static in vitro digestion) were analysed by HPLC-PDA-ESI-MSn and UHPLC-3Q-MS/MS. Broccoli stalks and co-products were featured by containing phenolic compounds (mainly hydroxycinnamic acid derivatives and glycosylated flavonols) and glucosinolates. The highest content of organosulfur compounds corresponding to the cores of the broccoli stalks treated by applying a drying descendant temperature gradient (aliphatic 18.05 g/kg dw and indolic 1.61 g/kg dw, on average, while the breakdown products were more abundant in the bark ongoing low temperature drying 11.29 g/kg dw, on average). On the other hand, for phenolics, feruloylquinic, and sinapoylquinic acid derivatives of complete broccoli stalk and bark, were more abundant when applying low-temperature drying (14.48 and 28.22 g/kg dw, on average, respectively), while higher concentrations were found in the core treated with decreasing temperature gradients (9.99 and 26.26 g/kg dw, on average, respectively). When analysing the bioaccessibility of these compounds, it was found that low-temperature stabilization of the core samples provided the material with the highest content of bioactives including antioxidant phenolics (13.6 and 33.9 g/kg dw of feruloylquinic and sinapoylquinic acids, on average, respectively) and sulforaphane (4.1 g/kg dw, on average). These processing options enabled us to obtain a new product or ingredient rich in bioactive and bioaccessible compounds based on broccoli stalks with the potential for antioxidant and anti-inflammatory capacities of interest.

## 1. Introduction

Broccoli (*Brassica oleracea* var. *italica*) awakened the interest of the scientific community and the consumers because of its bioactive phytochemical and nutritional wealth, including phenolic and organosulfur compounds, as well as essential vitamins and minerals [1]. However, the bioactive compounds are found not only in edible inflorescences or marketed heads but also in all the aboveground biomass (leaves and stalks) that represent up to 70% of the aerial parts of the broccoli plants [2]. This issue represents a real environmental problem that needs valorization alternatives to reduce the impact of the unused broccoli by-products to contribute to the sustainability of agricultural food production [3]. In other words, reducing the deleterious effects of the accumulation of agro-waste would help in the strategies of sustainability of the food production systems, contributing to the competitiveness of the producers and the circular economy of the food chains [4,5,6,7].

Beyond the potential uses identified, before planning the valorization alternatives, it is needed to fully characterize the material under study and, in this case, the phytochemical composition of broccoli stalks, which are characterized by a high content of (poly)phenols, glucosinolates, and isothiocyanates (GSL and ITC, respectively). Due to this composition, these by-products could represent a good source of functional ingredients [1,8,9]. The available experience demonstrated the capacity of this material to induce anti-cancer, anti-inflammatory, and/or modulatory effects on metabolic processes in different cell systems [5,10,11,12], focusing the attention on the organosulfur biocomponents to drive the fine-tuning of the processing methods [13]. The health-promoting activity is commonly ascribed, not only to a single compound, but also to a group of bioactive phytochemicals in the food matrix (e.g., kaempferol glycosides or hydroxycinnamic acid derivatives [14,15]) that may contribute to the capacity to scavenge reactive oxygen species (ROS) and, thus, reducing oxidative stress and mutagenic events [16]. Indeed, both types of compounds (organosulfur and phenolics) provide complementary bioactivities that boost the final health benefits of cruciferous foods [17]. Therefore, to take advantage of these biological functions, the adjustment of the processing protocols to gain a stable and safe product, maintaining the highest amount of original compounds possible, remains crucial [3]. Nonetheless, the content of bioactive compounds present in the stabilized plant material could not be the only criteria for decision-making, because the effects of gastrointestinal digestion to deliver the active fraction of the given phytochemical should be also taken into consideration [5,11,18,19,20].

To evaluate the effect of gastrointestinal digestion on the stability of broccoli phytochemicals and, thereby, on the biological interest of broccoli derived ingredients, the design of a proper processing method supported by sensitive analysis of phenolics and GSL/ITC is needed. Additionally, the study of the processing effects in combination with the physicochemical during the gastrointestinal digestion [13,17], would allow retrieving specific bioaccessible phytochemical profiles with multiple benefits on health [21].

Based on these premises, the present work pursues developing an efficient method to stabilize the phytochemical composition of the plant material (phenolics and other secondary metabolites and nutrients in agro-waste from broccoli), to maintain the highest bioaccessibility of bioactive compounds that would condition the biological impact. This fact supports the evaluation of the qualitative and quantitative composition of phenolic compounds and GSL after processing the broccoli’s stalks, together with the monitoring of the impact on their bioaccessibility. The resulting ingredient would open new opportunities for valorisation and recovery of agri-food waste for industrial applications.

## 2. Materials and Methods

### 2.1. Chemicals and Reagents

The standards of sinapic and caffeoylquinic acids were purchased from Sigma-Aldrich (Steinheim, Germany). The standards of glucoraphanin, glucoerucin, glucoiberin, glucobrassicin, hydroxyl-glucobrassicin, gluconasturtiin, methoxy-glucobrassicin, neoglubobrassicin, 3,4-diindolylmethane, and iberin (GR, GE, GI, GB, HO-GB, PE, MeOH-GB, NeoGB, SFN, I3C, and DIM, respectively) were purchased from Phytoplan GmbH (Heidelberg, Germany). The standards of sulforaphane, iberin, and indole-3-carbinol (SFN, IB, and I3C, respectively) were obtained from Santa Cruz Biotechnology (Dallas, US), Biorbyt LTD (Cambridge, UK), and LKT Laboratories (St. Paul, MN, USA), correspondingly. Acetic and hydrochloric acids, as well as ammonium acetate, were from Panreac labs (Barcelona, Spain). Methanol for hydromethanolic extractions and acetonitrile and acetic acid grade solvents for LC-MS were supplied by J.T. Baker (Philipsburg, NJ, USA). All water employed in the extraction and the chromatographic analysis was purified by using a Milli-Q system (Millipore, Bedford, MA, USA).

### 2.2. Plant Material and Processing Conditions

**Figure 1:**
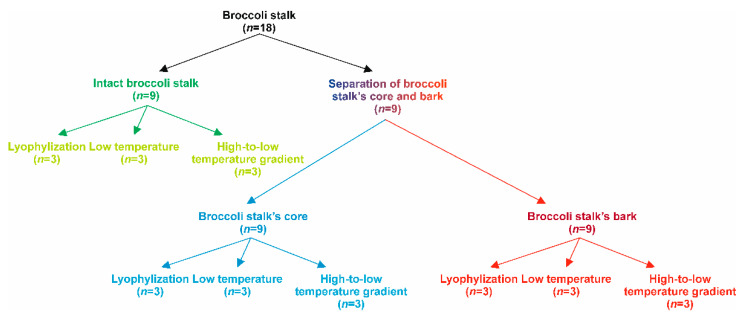
Schematic representation of the experimental design and processing scheme.

Broccoli plants (*Brassica oleracea* var. *Italica*, cv. ‘Parthenon’, Sakata Seeds Iberica, Valencia, Spain) were cultivated in the fall-winter cycle of 2021 under the conditions of the Semiarid Mediterranean climate of Murcia (SE Spain), in the experimental farm (La Matanza, CEBAS, Murcia) to reach the stage of harvest for commercial inflorescences or broccoli heads. Harvesting was performed when plants presented mature commercial flowering heads and their quality parameters (compactness, homogeneity of grain, absence of secondary heads, absence of minor leaves in the head, as well as the total absence of hollow stems) corresponded to ‘marketable’ class. All parameters were visually evaluated to establish that heads presenting more than two unacceptable physical parameters would be considered ‘non-marketable’. The inflorescences were separated from the stalks manually, and the broccoli stalks were collected and transferred to the laboratory (Phytochemistry and Healthy Food lab (LabFAS), CEBAS-CSIC, Murcia, Spain) for further processing. The period between sampling and processing was less than 4 h to avoid the degradation of compounds. Once in the lab, to set up the best processing conditions, the quantitative profiles of phenolic compounds, GSL, ITC, and indoles were determined in fresh broccoli stalks, as well as in the plant material exposed to diverse processing and stabilization options (Figure 1). In this regard, it is important stating that processing does not include only the time-temperature settings addressed to reduce the water and enzymatic activity of the plant material and thereby, the damaging reactions for the target compounds [22]. Pre-processing steps to separate broccoli stalk sub-fractions were also considered and monitored. So, the effect of the thermal treatments on the phytochemical load of the plant material was assessed not only on intact broccoli stalk but also on its core and bark considered separately. Based on these premises, the stalks were homogenously distributed before chopping each stalk or stem into 1 cm height discs. Again, all the discs were mixed thoroughly and bulked (*n* = 18). The discs of broccoli stalks were separated into two groups (Figure 1): One group of discs that would be thermally treated without additional manipulation (*n* = 9) and the second group of discs that would be processed by separating the bark (*n*= 9) from the core (*n* = 9). Then, intact discs, cores and barks samples were all submitted to the three treatments selected for stabilizing the composition: Lyophilisation or freeze-drying (−80 °C, *n* = 3), oven drying (40 °C for 72 h, *n* = 3), and descendent temperature gradient (Temp initial (75 °C)—temp final (60 °C) in 10 h, *n* = 3), aiming to dry the plant materials until constant weight that corresponded to losses of 83.0%, 92.7, and 98.3%, for intact broccoli stalks, core, and bark, respectively (Figure 1).

All materials (complete discs, cores, and barks of stalks) were used to prepare analytical extracts following three different procedures. For this purpose, samples (100 mg) were first homogenized in 1 mL of ethanol/deionized water (50:50, *v*/*v*) and then extracted (i) at 70 °C, for 20 min, with vortex shaking every 5 min. All the obtained extracts were centrifuged at 4000 rpm, for 5 min, filtered through a 0.22 μm PVDF filter (Millipore, MA, USA), and kept at −20 °C until chromatographic analysis [13,17].

### 2.3. In Vitro Simulated Gastrointestinal Digestion

Simulated gastrointestinal digestions were performed on brassica stalks powder following the methodology previously described [13]. Briefly, for the gastric digestions, the samples (500 mg) were mixed with 15 mL of simulated gastric fluid (SGF) stock electrolyte solution containing a mixture of salts simulating the composition of the SGF and the pepsin enzyme (EC 3.4.23.1) at the final concentration of 2000 U/mL. The final pH was adjusted to 3.0. The gastric digestion of the plant material in the SGF was performed for 2 h, at 37 °C, under continuous stirring. During incubation, the pH was double-checked every 15 min and corrected when necessary to ensure a constant pH of 3.0. The reaction was stopped by adding sodium hydroxide solution (0.2 M). To simulate intestinal digestions, the simulated intestinal fluid (SIF) was prepared by dissolving a mixture of salts in deionized water, as described in the literature [13]. On the prepared SIF, the enzymatic activity characteristic of intestinal digestion was achieved by mixing pancreatin (EC 232-468-9) and pancreatic lipase (EC 3.1.1.3) at the concentrations of 100 U/mL of trypsin activity and 64 U/mL of lipase activity for pancreatin and 2000 U/mL for pancreatic lipase. Frozen porcine bile salts were added to achieve the final concentration of 10 mM. The pH of the SIF was adjusted to 8.0. Again, the intestinal digestions were performed for 2 h at 37 °C in a thermal bath under continuous stirring [13].

After gastrointestinal digestion, the samples were centrifuged at 2000 rpm, for 5 min, at 4 °C to separate the soluble or bioaccessible fraction and the residual fraction. The bioaccessible fractions were filtered through a 0.22 μm PVDF filter (Millipore, MA, USA) and analysed by HPLC-PAD-ESI-MSn for phenolic compounds and GSL, and UHPLC-ESI-QqQ-MS/MS for ITC and nitriles.

### 2.4. HPLC-PAD-ESI-MSn Analysis of the Quantitative Glucosinolate and Phenolic Profiles

The chromatographic separation and spectrometry analysis of GSL and phenolic compounds present in the analytical extracts and gastrointestinal digestion products was performed following the methodology described by Baenas et al. and Abellán et al. [16,23]. The tentative identity of the phenolic compounds and GSL was attained according to the retention time (min), parent ions, and fragmentation patterns, in comparison with authentic standards and descriptions available in the literature [17] (Supplementary Tables S1 and S2). The quantification was performed on chromatograms recorded at 330 nm (phenolic acids) and 227 nm (GSL), applying calibration curves freshly prepared each day of analysis.

### 2.5. UHPLC-ESI-3Q-MS/MS Analysis of Glucosinolate’s Breakdown Products: Isothiocyanates and Indoles

The chromatographic separation of ITC and indoles present in the analytical extracts of broccoli and gastrointestinal digestion products was performed according to the methodology described by Domínguez-Perles et al., taking into consideration the modifications performed by Baenas et al. and Abellan et al. [11,13,24], using a UHPLC coupled with a 6460 triple quadrupole-MS/MS (Agilent Technologies, Waldbronn, Germany) and a Zorbax Eclipse Plus C18 column (2.1 × 50 mm, 1.7 µm). The identification of the ITC and indoles was based on their retention time, original mass, and specific fragmentation pattern in comparison with authentic standards, as well as with information retrieved from metabolomic databases and the literature [11,24] (Supplementary Table S3). The concentration of the compounds identified was calculated resorting to standard curves of authentic standards freshly prepared each day of analysis.

### 2.6. Statistical Analysis

Results are presented as means ± SD (*n* = 3). Before selecting the statistical test to be applied, the normal distribution of the results and the homogeneity of variance were assessed by Kolmogorov–Smirnov and Levene tests, respectively. Because of the normal distribution of the data, a one-way analysis of variance (ANOVA) was applied to compare >2 experimental conditions. When the ANOVA test informed on significant differences, Tukey’s multiple range tests were carried out. For the analysis of the different bioaccessibility provided by the separate processed materials, the final concentrations were compared by resorting to paired *t*-tests. The level of statistical significance was set at *p* < 0.05.

## 3. Results and Discussion

As referred to before, the present article pursues uncovering the influence of the stabilizing broccoli stalks regarding their quantitative profile of bioactive phytochemicals using different time-temperature conditions and, thus, identifying the best option to retrieve the highest amount of bioaccessible bioactive phenolics and organosulfur (ITC and nitriles) compounds. This issue deserves to be explored because, despite the cumulative evidence concerning the bioaccessibility of both types of bioactive compounds [13,17], there is no information on the relevance of the different starting phytochemical burdens to obtain significantly higher concentrations in the intestinal lumen available to be absorbed.

### 3.1. (Poly)Phenolic Content of Broccoli Stalk’s Fractions

The phenolic profile of broccoli stalks and fractions is represented by phenolic acids (Supplementary Table S1). The results obtained were fully coincident with PDA and spectrometric spectra recorded for authentic standards and the descriptions available in the literature [17,25,26]. This phenolic composition agrees with previous descriptions of broccoli that have shown higher levels of hydroxycinnamic acids (>92% of the total phenolic content), while the flavonoid derivatives are absent in broccoli by-products (leaves and stalks) [27].

All materials characterized in the present work presented a remarkable phenolic content, resulting from the combination of caffeoyl, feruloyl, coumaroyl, and sinapoyl derivatives (Table 1). The highest concentration for almost all phenolics identified corresponded to intact stalks and core that presented the following decreasing order sinapoyl derivatives (7.61–34.97 g/kg dw) > feruloyl derivatives (2.85–24.37 g/kg dw) > caffeoyl derivatives (1.17–7.90 g/kg dw) > coumaroyl derivatives (0.08–1.54 g/kg dw) (Table 1). In this regard, it should be clarified that although lyophilization is the standard pre-processing method to stabilize plant material and thereby, the reference considered in the present work for comparison, this process strongly affects the physical structure of the plant material influencing the occurrence of oxidation process [28]. This should be taken into consideration to fully understand the processes behind the values obtained for phytochemicals concentration.

The analysis of individual phenolics evidenced that the highest concentration of 5-caffeoylquinic acid corresponded to intact broccoli stalk and core, while it was at very low concentrations in bark (as for practically all phenolics). Thus, the concentration of 5-caffeoylquinic acid in the complete broccoli stalk and core. Other caffeoyl derivatives were found in a low concentration without a clear preponderance when comparing intact stalks and core. Additionally, the phenolic analysis allowed stressing the high amount of feruloyl-caffeoyl derivative corresponding to the transition *m*/*z* 551 and 469 arbitrary mass units (amu) to the fragments *m*/*z* 193, 275, 179 amu (Supplementary Table S1). These compounds were found in no significantly different concentrations in intact broccoli stalk and core, both of them exhibiting up to 86.4% higher concentrations than bark (Table 1). The most relevant differences between complete stalk and core were observed regarding caffeoyl-hexose derivative and 1,2,2′-tri-sinapoyl-gentibioside (both of them recorded at higher concentrations in complete broccoli stalks), as well as di-sinapoyl-gentibioside I and 1,2′-di-sinapoyl-gentibioside, the highest concentration of which corresponded to the core (Table 1).

The quantitative profile of phenolics in broccoli stalks and sub-tissues is in agreement with what was studied in broccoli by-products, also stabilized by applying lyophilization processes and, thereby, exposed to similar deleterious effects, where there is also a predominance of sinapic acid and derivatives, as well as, to a lesser extent, feruloyl derivatives [1,27]. This preponderance is of special relevance because of the strong radical scavenging activity demonstrated for sinapic acid derivatives, as well as their capacity to interact with the membrane lipids and, thus, participate in the prevention of lipid peroxidation [29,30]. These molecular capacities allow the envisaging of the application of the materials obtained as functional ingredients. Indeed, these results suggest that most compounds are in the core. In this regard, to the present date, it has been reported that specific phenolics could accumulate in particular tissues at particular times, while others could be considered almost ubiquitous [31]. Beyond the physiological facts governing the distribution of phenolics in specific tissues, cleaning up the broccoli stalks by removing the bark could give rise to new materials featured by specific composition (complex carbohydrates and/or lipoidal material) with positive/negative effects on the extractability of specific individual compounds [32].

### 3.2. Organosulfur Compounds of Broccoli Stalk, Core, and Bark

Beyond (poly)phenols, brassica foods are also characterized by the occurrence of typical organosulfur compounds (GSL, ITC, and nitriles), which develop biological functions complementary to those attributed to polyphenols (radical scavenging activity). So, once profiled the (poly)phenolic diversity of broccoli stalks, the plant materials obtained were also assessed on the content of individual GSL, ITC, and indoles (Table 2). These determinations provided the starting point of the plant material regarding the GSL profile, considered as a reference to recognize the effect of the stabilizing treatments thus, complementing the phytochemical picture of broccoli stalks. The identification of GSL in fresh and processed plant materials informed on quantitative profiles that were fully coincident with PDA and spectrometric spectra described in the literature [23].

The aliphatic GSL group of broccoli stalks were represented by GI, GR, and GE, the aromatic class by PE, and the indolic class by HGB, GB, MGB, and NGB, which is in good agreement with previous descriptions of the GSL profile of broccoli plant materials [5,6,10,13,21]. On the other hand, as expected, the ITC and indoles, which are produced as a result of the activity of the β-glucosidase myrosinase, when in contact with GSL upon physical disruption of the tissue [33], were almost absent in the lyophilized material. In this regard, low concentrations of SFN and I3C were detected, but still above the limit of quantification (LOQ) of the technique (signal to noise (S/N) ratio of 10:1 [34]).

The concentrations recorded in the present work for the individual GSL belonging to the separate classes were at similar levels to those previously reported concerning broccoli stalks, for instance, regarding GR, GE, GI, or GB (0.310–1.801, 0.095–0.141, and 0.535–2.024, and 0.280–0.357 mg/g dw, respectively) [1]. On the other hand, the modifications observed regarding the concentration of total and almost all individual GSL in the thermally dried materials (core and bark) would indicate, to some extent, that processing stalks in this way would facilitate the liberation of myrosinase to get into contact with GSL and triggering hydrolysis reactions to produce bioactive ITCs. This situation is suggested by the modification of the GSL concentration observed according to data in Table 2 and is essential to clarify how the stabilization of broccoli stalk, core, and bark, definitively modify their quantitative organosulfur profile and the consequence of such modifications for the bioaccessibility of the bioactive (broken-down) forms. Additionally, as referred to before, the impact that lyophilization exert on the physical properties of the plant material, and specifically on porosity could be shared to a different extent by other thermal treatments that, in turn, also would condition strongly the release of the target bioactive compounds during analytical extractions or as a result of the gastrointestinal digestion [28].

The plant materials assessed also exhibited the presence of GSL break-down products. These compounds were found at a higher concentration in the bark. This fact would be tentatively due to the effect of chopping broccoli stalks to separate core and bark, which could alter the integrity of the tissues and trigger the hydrolysis of GSL towards ITC and nitriles [33]. So, the content observed was at similar higher levels in complete broccoli stalks and bark that surpassed the concentration observed in the isolated core by 66.3%, on average. Additionally, as a result of the degradation of indolic GSL, I3C was found. This compound presented the highest concentration in bark, followed by core and complete broccoli stalk (79.2% and 74.6% lower, respectively) (Table 2). The additional breakdown products of GSL monitored in the present work (erucin, iberine, and DIM) were not found.

### 3.3. Effect of Processing Broccoli Stalk on the Phytochemical Composition

According to the characterization of the fresh materials described before, the broccoli stalk’s core appeared as the main source of most bioactive phytochemicals monitored (phenolic and organosulfur compounds). However, for the long-term use of such materials, they have to be stabilized by lowering the water content and, subsequently, the enzymatic activity and microbial growth. Different alternatives could be considered to achieve this goal. Deciding on the best choice would be based on the final concentration of phenolic and organosulfur compounds, as well as taking into consideration economic issues enclosed to the sustainability of the industrial activity that could be jeopardized by applying strict lyophilization processes. As described before, the production of bioactive ITC and nitriles depend on myrosinase activity, while the temperature conditions required to achieve this goal (>70 °C for >30 min) do not cause significant degradation of the phenolic fraction [35]. Testing both options is important because, to the present date, the best source of dietary organosulfur compounds to gain high amounts of bioaccessible ITC and nitriles remains unidentified and thus, conditions preserving the breakdown products of myrosinase were sought in the present work. Beyond the enzymatic activity, the differential effect of both treatments on the porosity of the processed plant material will be critical for obtaining high bioaccessibility rates [28]. Thereby, two drying conditions were assayed on the three materials to obtain stabilized co-products with a preserved (poly)phenolic fraction that were further assessed on the GSL, ITC, and nitriles, as complementary bioactive compounds to (poly)phenols.

#### 3.3.1. Modification of the Quantitative (Poly)Phenolic Profile

The assessment of the (poly)phenolic burden of these materials allowed obtaining a complete picture of the effect of processing on the functional scope of phytochemical composition of broccoli stalks and co-products (Table 3).

It should be stressed that (poly)phenols are thermolabile compounds although not all phenolic sub-classes to the same extent [36]. In this regard, the phenolic fraction of broccoli stalks is mainly represented by caffeoyl, feruloyl, and sinapoyl derivatives (Table 1). These groups of phenolics followed an equal trend when exposed to the low-temperature and time-temperature gradient drying conditions (Table 3).

Concerning caffeoyl/feruloyl derivatives, despite the higher concentration observed in complete stalk and core relative to bark, after drying these differences almost disappeared since the formers decreased the total concentration up to 55.1%, depending on the stabilizing treatment and the bark augment the content of feruloyl/caffeoyl derivatives 3.0 folds, on average. In this regard, beyond the comparison of materials, it was found that drying the separate materials by applying low temperatures allowed for obtaining significantly higher concentrations of caffeoyl/feruloyl derivatives relative to the application of time-temperature gradients (Table 3).

This finding is in agreement with previous descriptions of the deleterious effect of high temperature on phenolic acids that cause losses of up to 87% [37]. However, in the present work, the decrease observed was lower (<55.1%), which could be due to the thermal treatments applied. The reductions described by Vallejo et al. were due to the application of four domestic cooking processes featured by moist heat (high-pressure boiling, low-pressure boiling (conventional), steaming, and microwaving were the four domestic cooking processes), thus describing high rates of compounds leaching to the medium beyond their thermal degradation. This contrasts with the dry heat applied in the present work. The separate individual caffeoyl/feruloyl acids exhibited different sensitivity to the stabilization conditions. Although the concentration of compounds within this group decreased due to the thermal treatments, di-caffeoylquinic acid augmented its concentrations in nearly all materials and treatments (except for complete broccoli stalks suffering time-temperature drying) (Table 3). This different trend would be associated with the nature of phenolics, which may readily interact with each other to yield complexes in a wide range of food systems, being temperature the most critical factor [38]. This is relevant because the production of specific chemical forms is not only resulting from the interaction between phenolics but also with other complex molecules, such as proteins or carbohydrates, which impacts the bioaccessibility and biological activity (functionality and nutraceutical properties) of the phenolic fraction [39]. These interactions should be tailored to develop food products and ingredients with maximized functionality and quality attributes [38].

Concerning sinapoyl derivatives, decreased burdens in complete broccoli stalks and core, as well as augmented concentrations in processed bark, were found, while the height of the modifications observed was dependent on the material considered. So, the lowest changes corresponded to complete broccoli stalks on which no significant differences between lyophilized and processed materials were observed (Table 3). The core exhibited higher losses of sinapoyl derivatives in materials stabilized by applying low-temperature drying than in the exposed to time-temperature gradients (60.8% and 20.0%, respectively) and bark displayed higher concentrations of this type of phenolics in materials dried by low-temperature conditions relative to the time-temperature gradient (by 6.6-fold, on average). Concerning the individual sinapoyl derivatives, as referred to for other phenolic classes, almost all individual sinapoyl-based compounds identified reduced the concentration in the stabilized materials as a consequence of the drying process, except for di-sinapoyl-di-glucoside, which augmented its concentration relative to the fresh material by low-temperature drying (2.3-fold on average in the three materials) and time-temperature gradient (4.7-fold, on average, in the three materials) (Table 3).

The different trends observed regarding the final concentration of the diverse individual caffeoyl/feruloyl and sinapoyl derivatives trend would be associated with the nature of phenolics, which may readily interact with each other to yield complexes in a wide range of food systems, being temperature a critical factor for these reactions [38]. This is relevant in the frame of the present work because the production of specific chemical forms not only resulting from the interaction between phenolics but also with other complex molecules, such as proteins or carbohydrates, impacts directly on the bioaccessibility of the phenolic fraction and, thus, on the biological activity (functionality and nutraceutical properties) expected that has been described as closely related to the chemical structure [39]. These interactions should be tailored to develop food products and ingredients with maximized functionality and quality attributes [38]. Additionally, the effect of the thermal treatment on the physical structure of the plant material is responsible for the different behaviour observed for complete broccoli stalks, core, and bark.

#### 3.3.2. Modification of the Quantitative Profile of Organosulfur Compounds

The joint assessment of the organosulfur compounds in the processed materials complemented in a valuable way the (poly)phenolic characterizations described in the previous section. So, after processing by applying low temperatures, GI, GR, GE, and PE did not exhibit significant differences between matrices (complete stalks, core, and bark) because this drying condition augmented 2-fold the concentration of aliphatic and aromatic GSL (Table 4).

Nonetheless, interestingly, when applying the decreasing temperature gradient, a significant increase in this GSL fraction was observed in the core (almost 7-fold augment) allowing obtaining a material with a significantly higher concentration of GSL (*p* < 0.001) relative to complete stalks and bark. The relative abundance of the individual GSL was not modified significantly, appearing GR as the most abundant one in all samples (54.5–67.3% of aliphatic and indolic GSL). In addition, it was stressed the presence of GI and PE, which relative abundance varied depending on the material considered. So, as a general trend, while in the complete stalks and core exposed to low temperature drying GI was more abundant than PE (17.6% and 10.5%, respectively), this trend was reversed when applying the time-temperature drying gradient (10.7% and 18.3%, correspondingly) (Table 4).

Concerning indole GSL, it was observed the presence of HGM, GB, MGB, and NGB. However, for this class, differences between materials and due to the different processing conditions were recorded. Thus, for complete broccolis stalks, the concentration of indole GSL remained almost unaltered after both drying treatments, while the application of low-temperature for drying caused an increased concentration of total indole GSL of both core and bark (by 17.4% and 126.1%, correspondingly) (Table 4).

Interestingly, the application of the decreasing temperature gradient displayed different effects on the core and bark. Although, in the former, this stabilizing method entailed a 3.2-fold increase in the indole GSL content, on average, relative to both fresh material and core exposed to low-temperature drying, the application of the same conditions to bark caused a very limited augment in comparison with fresh material (1.2%) and an almost 2-fold lower concentration relative to the low-temperature drying (Table 4).

The analysis of the relative contribution of the individual indole GSL evidenced differences mainly due to the type of material and, to a lesser extent, the drying method. So, in general, MGB was the most abundant compound in both complete stalks and core after stabilization applying low-temperature (51.0–65.4%) while for core exposed to the time-temperature gradient and bark, NGB was the most abundant compound (36.9–48.0%). Additionally, a high concentration of GB was recorded in all materials that ranged between 11.3% (low-temperature dried core) and 30.0% (time-temperature dried core). Finally, although HGB was detected in all materials the stabilization processes significantly reduced its concentration, being the time-temperature gradient more destructive for this GSL (Table 4).

Concerning ITC and indoles, SFN and I3C were the only compounds identified in all samples. The latter was the most abundant with percentages in the range of 82.6–96.4% (Table 4). For all materials, the low-temperature processing was associated with an increase in the GSL breakdown products, mainly due to the augment of I3C (SFN appeared as a more stable compound), although this increase was only significant for complete stalks and bark (Table 4). Interestingly, the application of the decreasing temperature gradient displayed. Additionally, for both complete broccoli stalks and bark, the gradient drying significantly reduced the indole content; while for core, this treatment entailed a significant augment relative to fresh and low-temperature processed materials (63.4% and 46.7% lower, respectively).

In a previous experience, it was observed that air drying at 40 °C does not impact significantly the total amount of GSL of broccoli by-products [40], which are in good agreement with the limited variations obtained in the present work when drying by applying low temperatures. In this regard, these processing conditions do not inactivate myrosinase [33], and, thereby, could cause the hydrolysis of GSL during the analytical extraction, no significant modifications were observed that could be due to a reduced release from the plant material the structure of which seems to be highly preserved as a result of the referred to stabilizing treatment. Indeed this (the effect on the physical structure of the plant material) constitutes a critical difference between stabilization methods [28]. Additionally, it has been reported that these compounds remain at similar levels when applying temperatures of up to 70 °C for 20 min [41]. In this sense, and according to the time-temperature gradient applied, it seems that high temperatures during longer periods (>20 min) could inactivate myrosinase, leading to higher concentrations of GSL, especially in broccoli stalks’ core [33]. At this point, the selection of the best option depends, not only on the quantitative phytochemical profile of the plant material but also on the capacity of the gastrointestinal digestion to extract the target bioactive organosulfur compounds from the materials obtained.

### 3.4. Bioaccessibility of Phenolic Compounds and Glucosinolates from the Different Matrices

Despite the valuable content of both phenolic and organosulfur compounds in fresh and processed broccoli stalks described in the previous sections, to identify the best dietary source of bioactive phenolics, ITC, and nitriles, all these matrices need to be explored regarding the effect of the gastrointestinal digestion on the stability of the target phytochemicals. This approach was envisaged to provide valuable information for the selection of the best processing alternative to retrieve the highest bioaccessibility and, thus, take advantage of the biological functions of broccoli stalk phytochemicals.

**Figure 2:**
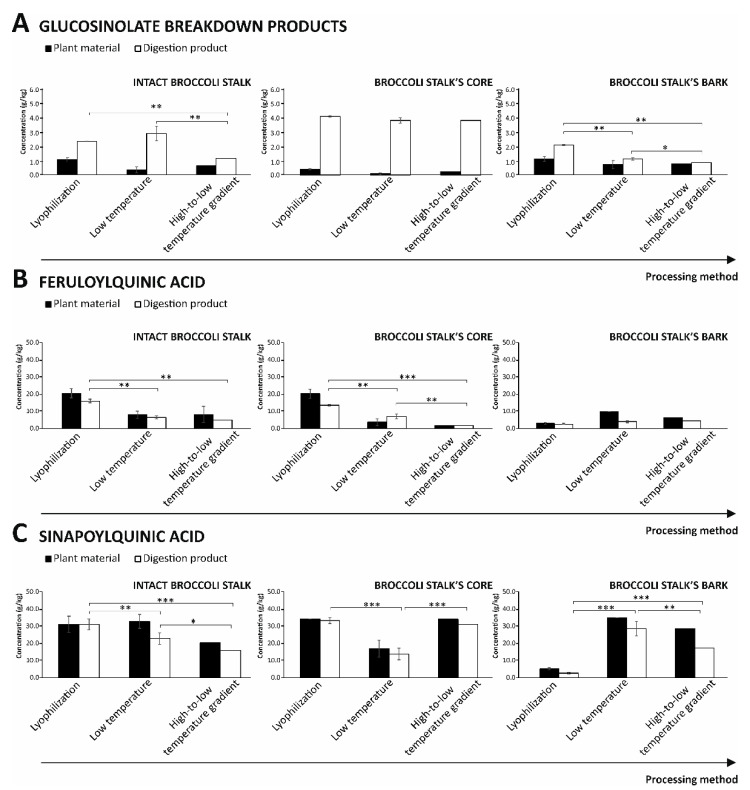
Content of glucosinolates breakdown products (**A**), feruloylquinic acids (**B**), and sinapoylquinic acids (**C**) of in vitro gastrointestinal digestion products of lyophilized, low-temperature dried, and high-to-low temperature gradient dried broccoli stalks and co-products. Significant differences were according to a paired *t*-test (*p* < 0.05 (*), *p* < 0.01 (**), and *p* < 0.001 (***)).

For this purpose, the quantification of the bioactive metabolites obtained during the gastrointestinal digestion of broccoli stalks undergoing freeze-drying and drying by applying a low temperature or high-to-low temperature gradient was developed (Figure 2).

The analysis of the concentration of feruloylquinic and sinapoylquinic acids in the digestion products evidenced that almost all treatments entailed a degradation of the phenolic fraction that remained in the rages of 1.8–13.6 mg/kg dw and 14.0–33.9 mg/kg dw for feruloylquinic (represented by 5-caffeoylquinic acid and p-coumaroylquinic acid) and sinapoylquinic (represented by di-sinapoyl glucoside) acids, respectively (Figure 2). According to these results, the final concentration achieved for cores processed applying low-temperature drying provides 4.3-fold higher and 2.3-fold lower amounts of feruoylquinic and sinapoylquinic acids, respectively. Because of the lower concentration of feruloylquinic acids in the digestive products, this is the limiting factor that would indicate the capacity of the low-temperature process of the broccoli stalk’s core to retrieve the utmost quantitative profile of bioactive phytochemicals. This content is of special interest given the radical scavenging activity featuring phenolics with special emphasis on the prevention of lipid peroxidation [30,42]. These scavenging functions constitute a valuable contribution to the biological functions attributed to the organosulfur compounds (mainly anti-inflammatory and anti-tumoral) since the reduction in the reactive oxygen species in affected tissues would prevent the triggering of genotoxic and mutagenic effects that jeopardize the cellular functions [43].

The data retrieved evidenced that no GSL was found in the digestive products being all them breakdown towards specific breakdown products (ITCs and nitriles) among which only SFN was found in concentrations higher than the LOQ of the analytical technique mainly due to hydrolytic reactions during digestion [13,34]. This metabolite has been broadly characterized revealing cutting edge information on its health benefits, specifically related to preventing the onset of diverse types of cancer [44], in addition to key physiological activities, such as anti-depressant/anxiolytic-like effects, hypoglycemic, and anti-inflammatory activities [45,46,47]. Related to the latter activity and closely related to the concentration of dietary SFN reached in the upper intestinal sections, SFN has been recently related to the prevention of the severity of intestinal bowel disease, an emerging epidemic disorder that needs new therapeutic agents contributing to lower the incidence and severity of the clinical symptoms [48]. In this frame, the current study showed that although all materials digested after processing by lyophilisation, low-temperature, and decreasing temperature gradient drying experienced an augment of the SFN concentration in the digestion process, the highest values were for broccoli stalk’s core independently of the treatment applied, achieving concentrations of 3.8–4.1 g/kg dw (Figure 2). Thereby, this result points out further processing of broccoli stalks to remove bark and thus, enhance the extraction and hydrolysis of GR and finally the concentration of SFN in the intestinal lumen. For the selection of the best stabilization process attention should be paid to the phenolic fraction.

## 4. Conclusions

Broccoli stalks have been largely promoted as a sustainable source of bioactive phytochemicals with great potential for human health protection after dietary ingestion as a food ingredient or nutraceutical. However, these biological properties have been based on the quantitative phytochemical profile of intact materials. To transfer this hypothesis to practical applications and biological advantages actions are needed: gather information concerning the effect of the plant material processing on the phytochemical profile and the final bioaccessibility of bioactive compounds featuring broccoli. These gaps of knowledge were covered by the present work, providing evidence of the advantageous application of low-temperature stabilization that has been demonstrated in the present work as capable to preserve the high amount of bioaccessible SFN, while minimizing the loss of phenolic compounds, on which no significant differences were found relative to the plant material. As a result, the processes described in the present work allow obtaining a broccoli stalk-based material with promising phytochemicals composition and release/stability during the gastrointestinal digestion, providing low-temperature processing with the utmost intestinal concentration of bioactive compounds. The characterization of the actual functionality of the concentrations reached after digestion according to the anti-inflammatory activity and prevention of oxidative stress will give rise to their utilization in the prevention/treatment of specific health disturbances. Nonetheless, the methodology described continues to need additional clarifications to fine-tuning the uses of the intermediate products obtained. So, the product obtained would be of direct application for the design of nutraceutical products that already could be evaluated by resorting to pre-clinical and clinical research models of digestion, bioaccessibility, and bioavailability, as well as to mechanistic studies of functionality in vitro, mainly related to inflammation or para-inflammation. On the other hand, the inclusion of this intermediate product as an ingredient in the development of new functionalized foods entails the requirement of new determinations to understand the extent, to which the manufacturing processes could affect the stability and/or bioaccessibility of the target bioactive phytochemicals.

## Figures and Tables

**Table 1:** Content (g/kg dw) of individual glucosinolates and breakdown products in lyophilized broccoli stalks fractions (intact stalks, core, and barks).

Compound	Intact Broccoli Stalk	Broccoli Stalk’s Core	Broccoli Stalk’s Bark	*p*-Value
5-caffeoylquinic acid	4.30 ± 0.24 b	4.36 ± 1.01 b	0.06 ± 0.02 a	***
Caffeoyl derivative	1.73 ± 0.23 ab	2.24 ± 0.65 b	1.00 ± 0.03 a	*
Caffeoyl-hexose derivative	1.87 ± 0.24 c	0.72 ± 0.14 b	0.11 ± 0.02 a	***
*p*-coumaroylquinic acid	0.55 ± 0.01 b	1.54 ± 0.21 c	0.08 ± 0.01 a	***
Sinapoyl-gentibioside	0.85 ± 0.03 b	1.98 ± 0.42 c	0.08 ± 0.01 a	***
Sinapoyl hexoside	0.59 ± 0.10 b	0.77 ± 0.19 b	0.03 ± 0.01 a	**
Feruloyl-caffeoyl derivative	4.89 ± 0.83 b	3.72 ± 0.87 b	1.80 ± 0.09 a	**
Di-sinapoyl-gentiobioside I	0.70 ± 0.10 a	5.06 ± 0.53 b	0.78 ± 0.06 a	***
3-*O*-feruloylquinic acid	0.82 ± 0.23 b	1.12 ± 0.23 b	0.05 ± 0.02 a	**
Feruloyl-caffeoyl derivative	6.45 ± 0.8 b	7.37 ± 1.60 b	1.00 ± 0.08 a	***
Di-sinapoyl-diglucose	2.26 ± 0.10	4.37 ± 0.89	3.15 ± 0.02	N.s.
Di-caffeoylquinic acid derivative	1.76 ± 1.30	2.44 ± 0.53	1.58 ± 0.01	N.s.
Di-sinapoyl-gentiobioside II	4.69 ± 0.20 b	6.23 ± 0.97 b	0.72 ± 0.01 a	***
1-Di-sinapoyl-2-feruloyl-gentiobioside	0.70 ± 0.07 b	1.00 ± 0.21 b	0.21 ± 0.01 a	***
1-Di-sinapoyl-2-feruloyl-gentiobioside (isomer)	1.25 ± 0.05 b	1.40 ± 0.25 b	0.16 ± 0.02 a	***
1,2,2′-Tri-sinapoyl-gentiobioside	12.70 ± 0.12 c	8.77 ± 0.65 b	0.73 ± 0.02 a	***
1,2′-Di-sinapoyl-2-feruloyl-gentiobioside	1.94 ± 0.04 b	2.95 ± 0.68 c	0.17 ± 0.01 a	***

Mean ± SD (*n* = 3) followed by different lowercase letters are significantly different according to a one-way analysis of variance (ANOVA) and Tukey’s multiple range test. ESI, electrospray ionization; MRM, multiple reaction monitoring; N.s., not significant; *p* < 0.001 (***), *p* < 0.01 (**), and *p* < 0.05 (*).

**Table 2:** Content (g/kg dw) of individual glucosinolates and breakdown products of fresh broccoli stalks fractions (intact stalks, core, and bark).

Compound	Intact Broccoli Stalk	Broccoli Stalk’s Core	Broccoli Stalk’s Bark	*p*-Value
Aliphatic glucosinolates				
Glucoiberin (GI)	0.403 ± 0.083 a	0.269 ± 0.038 a	0.762 ± 0.025 b	***
Glucoraphanin (GR)	2.634 ± 0.691 b	1.273 ± 0.144 a	0.185 ± 0.091 a	**
Glucoerucin (GE)	0.227 ± 0.046 a	0.125 ± 0.012 a	0.570 ± 0.096 b	***
Indolic glucosinolates				
Hydroxy-glucobrassicin (HGB)	0.108 ± 0.013 b	0.043 ± 0.007 a	0.092 ± 0.006 b	***
Glucobrassicin (GB)	0.110 ± 0.023 b	0.100 ± 0.002 b	0.046 ± 0.010 a	**
Methoxy-glucobrassicin (MGB)	0.449 ± 0.067 c	0.099 ± 0.012 a	0.182 ± 0.006 b	***
Neo-Glucobrassicin (NGB)	0.091 ± 0.014 a	0.216 ± 0.047 b	0.141 ± 0.012 a	**
Aromaticc glucosinolate				
Gluconasturtiin (PE)	0.652 ± 0.093	0.673 ± 0.112	0.510 ± 0.064	N.s.
Isothyocianates and indoles				
Sulforaphane (SFN)	0.91 ± 0.16 b	0.31 ± 0.05 a	0.92 ± 0.14 b	**
Indole-3-carbinol (I3C)	1.87 ± 0.32 a	2.27 ± 0.15 a	7.36 ± 0.76 b	***

Mean ± SD (*n* = 3) followed by different lowercase letters are significantly different according to a one-way analysis of variance (ANOVA) and Tukey’s multiple range test. N.s., not significant; *p* < 0.001 (***) and *p* < 0.01 (**).

**Table 3:** Phenolic content (mg/kg dw) of fresh, standard-dry, and gradient-dry intact broccoli stalks, broccoli stalk’s core, and broccoli stalk’s bark.

Compound	Intact Broccoli Stalk				Core				Bark				Comparison of Materials (LSD *p* < 0.05)		
	Lyo	LT	Grad	LSD (*p* < 0.05)	Lyo	LT	Grad	LSD (*p* < 0.05)	Lyo	LT	Grad	LSD (*p* < 0.05)	Lyo	LT	Grad
Caffeoylquinic acid derivatives															
5-caffeoylquinic acid	4.30 B c	1.89 C b	0.46 A a	0.49	4.36 B b	0.23 B a	<LOQ A a	1.17	0.12 A a	<LOQ A a	<LOQ A a	0.14	1.19	0.53	0.43
Caffeoyl derivative	1.73 AB b	0.46 A a	0.69 B a	0.13	2.24 B b	0.37 A a	<LOQ A a	0.76	0.71 A a	1.45 B a	0.77 B a	0.67	0.96	0.33	0.14
Caffeoyl-hexose derivative	1.87 C c	0.50 A b	0.27 B a	0.36	0.42 B b	0.42 A b	<LOQ A a	0.20	0.01 A a	0.33 A b	0.20 B b	0.18	0.20	0.40	0.09
*p*-coumaroylquinic acid	0.55 B c	0.38 A b	0.09 B a	0.06	1.03 C b	1.12 B b	<LOQ A a	0.21	0.06 A a	0.37 B c	0.20 C b	0.06	0.53	0.14	<0.01
Feruloyl-caffeoyl derivative	4.89 B c	0.46 A a	3.87 B b	1.07	3.72 B b	0.98 B a	1.20 A a	1.01	1.20 A a	2.36 C c	1.05 A b	0.19	1.07	0.17	1.02
3-*O*-feruloylquinic acid	0.63 B b	0.43 B a	0.38 B a	0.11	1.12 B b	0.04 A a	0.06 A a	0.26	0.03 A a	0.57 B b	0.56 C b	0.14	0.29	0.40	0.40
Feruloyl-caffeoyl derivative	6.45 B c	3.84 B b	1.02 A a	0.59	7.37 B b	0.56 A a	0.42 A a	1.85	1.00 A a	4.78 C b	3.75 B b	1.11	1.87	0.43	1.16
Di-caffeoylquinic acid derivative	3.06 B b	6.59 B c	<LOQ A a	0.14	2.01 A a	4.29 A a	8.31 C b	2.13	1.05 A a	4.56 A a	4.04 B a	3.31	0.50	2.46	3.03
Sinapoyl derivatives															
Sinapoyl-gentibioside	0.05 A a	0.95 B b	0.05 A a	0.26	1.16 B c	0.87 B b	0.53 C a	0.12	0.05 A a	<LOQ A a	0.38 B b	0.06	0.06	0.36	<0.01
Sinapoyl hexoside	0.59 B ab	0.52 A a	0.65 B b	0.16	0.77 B b	0.48 A ab	0.21 A a	0.25	0.03 A a	0.79 B b	0.88 B b	0.17	0.22	0.21	0.17
Di-sinapoyl-gentiobioside I	0.70 A a	1.73 B b	5.17 B c	0.77	4.41 B b	0.99 A a	1.63 A a	1.39	0.52 A a	1.11 A a	2.05 A b	0.41	1.38	0.28	0.85
Di-sinapoyl-diglucose	3.89 AB a	9.07 B b	14.93 A c	2.95	4.37 B a	8.13 B b	21.13 B c	2.68	2.10 A a	5.58 A b	11.96 A c	1.95	1.25	1.50	3.99
Di-sinapoyl-gentiobioside II	4.69 B b	5.17 B b	1.01 A a	0.48	6.23 B b	1.05 A a	0.93 A a	1.16	0.48 A a	7.19 C c	2.66 B b	1.01	1.13	0.88	0.74
1-Di-sinapoyl-2-feruloyl-gentiobioside	1.96 B b	1.50 B ab	0.70 A a	0.77	2.72 B b	0.46 A a	0.42 A a	0.92	0.25 A a	2.55 C c	1.38 B b	0.36	0.90	0.62	0.62
s	2.58 B c	1.48 B b	0.22 A a	0.06	2.95 B a	0.25 A a	0.71 A a	1.88	0.14 A a	2.50 C c	1.01 A b	0.22	0.69	0.13	1.79
1,2′-Di-sinapoyl-2-feruloyl-gentiobioside	27.96 B b	26.00 B ab	23.85 A a	1.52	32.84 B c	12.87 A a	26.26 A b	2.95	4.20 A a	30.44 C c	24.40 A b	2.20	3.00	0.86	2.07

Bars represent mean ± SD (*n* = 3). Bars with different lowercase letters are significantly different at *p* < 0.05 according to a one-way analysis of variance (ANOVA) and a multiple range test of Tukey. Lyo, lyophilization; LT, low-temperature drying; Grad, Decreasing temperature gradient. Different lowercase letters indicate significant differences between processing conditions for the same material and distinct capital letters indicate differences between materials for different processing conditions.

**Table 4:** Content of organosulfur compounds (mg/kg dw) of fresh, standard-dry, and gradient-dry intact broccoli stalks, broccoli stalk’s core, and broccoli stalk’s bark.

Compound	Intact Broccoli Stalk				Core				Bark				Comparison of Materials (LSD *p* < 0.05)		
	Lyo	LT	Grad	LSD (*p* < 0.05)	Lyo	LT	Grad	LSD (*p* < 0.05)	Lyo	LT	Grad	LSD (*p* < 0.05)	Lyo	LT	Grad
Aliphatic and aromatic glucosinolates															
Gluciberin	403.02 B a	566.64 B b	407.99 A a	123.58	268.87 A a	823.08 C b	1871.79 B c	373.42	762.23 C b	241.46 A a	226.10 A a	50.98	79.89	140.78	362.09
Glucoraphanin	2633.89 B a	2212.52 AB a	2157.00 A a	675.54	1272.52 A a	2683.17 B b	11,447.19 B c	845.76	1184.84 A a	2021.38 A c	1655.10 A b	254.50	597.15	431.46	832.88
Glucoerucin	277.30 A a	244.20 B a	280.28 A a	56.86	125.45 A a	395.04 C a	1821.21 B b	401.50	570.28 B b	134.40 A a	138.82 A a	115.16	117.95	55.63	400.86
Gluconasturtin	652.00 A b	375.36 A a	740.53 A b	155.77	672.77 A a	435.58 AB a	2910.78 B b	385.59	510.22 A a	559.30 C ab	771.34 A b	192.02	158.91	116.28	413.58
Indolic glucosinolates															
Hydroxy-glucobrassicin	107.91 B b	<LOQ A a	<LOQ A a	10.65	43.33 A b	59.86 B c	<LOQ A a	13.12	91.76 B a	66.95 B a	74.44 B a	24.28	15.13	13.76	21.33
Glucobrassicin	109.81 B b	113.27 B b	57.92 A a	32.98	100.47 B a	60.94 A a	481.77 B b	54.53	46.19 A a	193.26 C c	89.98 A b	27.43	29.51	26.68	56.84
Methoxy-glucobrassicin	449.05 A b	416.89 B ab	327.92 B a	78.57	99.47 C a	275.13 A b	496.99 C c	37.76	182.17 B a	279.09 A b	155.05 A a	29.14	55.66	43.28	58.97
Neoglucobrassicin	90.73 A a	106.94 A a	168.13 A b	22.77	215.96 B a	143.64 A a	626.96 B b	80.69	140.97 AB a	497.12 B b	186.63 A a	81.12	75.16	72.01	58.67
Isothiocyanates and indoles															
Sulforaphane	1.09 B c	0.35 A a	0.62 B b	0.16	0.41 A c	0.10 A a	0.23 A b	0.06	1.10 B a	0.71 A a	0.77 C a	47.48	0.24	0.48	0.08
Erucin	<LOQ	<LOQ	<LOQ	N.d.	<LOQ	<LOQ	<LOQ	N.d.	<LOQ	<LOQ	<LOQ	N.d.	N.d.	N.d.	N.d.
Indole-3-carbinol	1.50 A b	3.95 A c	<LOQ A a	1.13	1.94 A a	3.33 A b	6.20 C c	0.85	7.48 B b	10.58 B c	4.94 B a	1.26	1.01	1.46	0.65

Bars represent mean ± SD (*n* = 3). Bars with different lowercase letters are significantly different at *p* < 0.05 according to a one-way analysis of variance (ANOVA) and a multiple range test of Tukey. Lyo, lyophilization; LT, low-temperature drying; Grad, Decreasing temperature gradient. Different lowercase letters indicate significant differences between processing conditions for the same material and distinct capital letters indicate differences between materials for different processing conditions. N.d., not determined.

## Data Availability

The data are available from the corresponding author.

## Supplementary Materials

The following supporting information can be downloaded at: https://www.mdpi.com/article/10.3390/foods11121734/s1, Table S1: Qualitative HPLC-PDA-ESI/MSn analysis of the individual phenolic compounds present in analytical extracts and digestion products of pre-processed broccoli (Brassica olreacea var. itálica) stalks; Table S2: Qualitative HPLC-PDA-ESI/MSn analysis of the aliphatic, aromatic, and indolic glucosinolates present in analytical extracts and digestion products of pre-processed broccoli (Brassica olreacea var. itálica) stalks; Table S3: Fragmentation patterns monitored by UHPLC-ESI-QqQ-MS/MS for the identification and quantification of glucosinolates breakdown products present in analytical extracts and digestion products of pre-processed broccoli (Brassica olreacea var. itálica) stalks.
