# Supplementary material for: A New Food Ingredient Rich in Bioaccessible (Poly)Phenols (and Glucosinolates) Obtained from Stabilized Broccoli Stalks
Source: Foods. 2022 Jun 14;11(12):1734. doi: 10.3390/foods11121734 (PMC9222756; doi:10.3390/foods11121734)
Supplement: Supplementary file 1 [file foods-11-01734-s001.zip › foods-1736046-supplementary.pdf]

Supplementary Table S1. Qualitative HPLC-PDA-ESI/MSn analysis of the individual phenolic compounds present in analytical extracts and digestion products of pre-processed broccoli (*Brassica olreacea* var. *itálica*) stalks.

Representative HPLC-PDA-ESI-MSn chromatogram of the intact broccoli stalks (black line), broccoli stalk's core (blue line), and broccoli stalk's bark (red line) recorded and 330 nm.

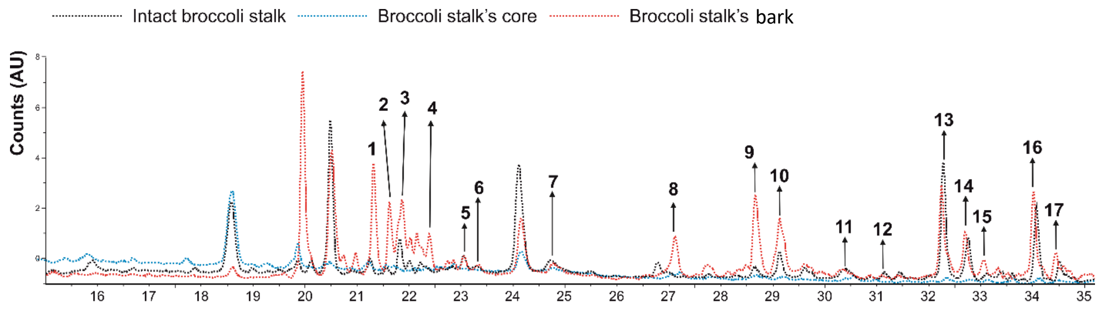

Peak nº	Compound	Retention time (min)	Parent Ion (*m/z* [M-H])	Product Ions (*m/z* MSn[M-H])
1	5-caffeoylquinic acid	21.4	353	191,179
2	Caffeoyl derivative	21.6	306	253,179
3	Caffeoyl-hexose derivative	21.9	421	341,179
4	*p*-coumaroylquinic acid	22.4	337	163,191
5	Synapoyl-gentibioside	23.0	547	223,163
6	Synapoyl hexoside	23.3	385	223,163
7	Feruloyl-caffeoyl derivative	24.8	551	193,275,179
8	Di-sinapoyl-gentiobioside I	27.1	753	529
9	3-*O*-feruloylquinic acid	28.8	885	723,499
10	Feruloyl-caffeoyl derivative	29.2	469	193,275,179
11	Di-sinapoyl-diglucose	30.4	753	591
12	Di-caffeoylquinic acid derivative	31.2	739	515,275
13	Di-sinapoyl-gentibioside II	32.3	753	529
14	1-di-sinapoyl-2-feruloyl-gentiobioside	32.8	723	499,223
15	1-di-sinapoyl-2-feruloyl-gentiobioside (isomer)	33.1	723	499,223
16	1,2,2'-tri-sinapoyl-gentiobioside	34.2	959	735, 205,511,529
17	1,2'-di-sinapoyl-2-feruloyl-gentiobioside	34.6	929	705,511,222,529

Supplementary Table S2. Qualitative HPLC-PDA-ESI/MSn analysis of the aliphatic, aromatic, and indolic glucosinolates present in analytical extracts and digestion products of pre-processed broccoli (*Brassica olreacea* var. *itálica*) stalks.

Representative HPLC-PDA-ESI-MSn chromatogram of the intact broccoli stalks (black line), broccoli stalk's core (blue line), and broccoli stalk's bark (red line) recorded and 227 nm.

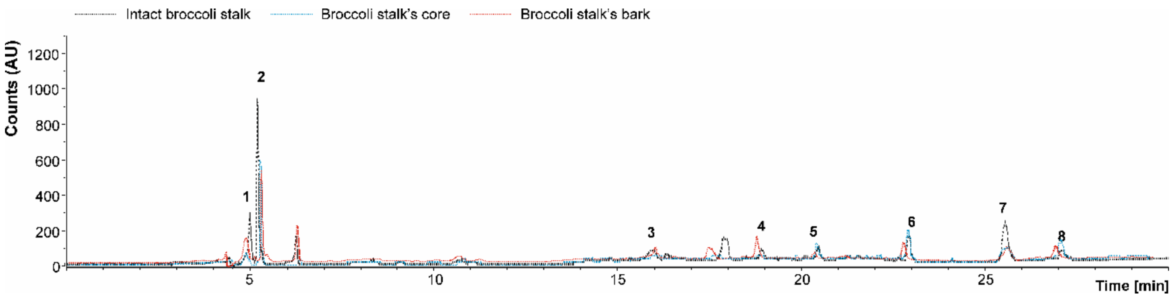

Peak nº	Compound	Glucosinolate type	Retention time (min)	Parent Ion (*m/z* [M-H])	Product Ions (*m/z* MSn[M-H])
1	Glucoiberin (GI)	Aliphatic	5.3	422	259, 97
2	Glucoraphanin (GR)	Aliphatic	6.0	436	372,259,97
3	Hydroxy-glucobrassicin HGB)	Indolic	16.2	463	285,241,97
4	Glucoerucin (GE)	Aliphatic	18.9	420	259,97
5	Glucobrassicin (GB)	Indolic	20.5	447	404,259,97
6	Gluconasturtiin (PE)	Aromatic	23.0	422	259,97
7	Methoxy-glucobrassicin (MGB)	Indolic	25.6	477	259,97
8	Neo-Glucobrassicin (NGB)	Indolic	27.8	477	446,259,97

Supplementary Table S3. Fragmentation patterns monitored by UHPLC-ESI-QqQ-MS/MS for the identification and quantification of glucosinolates breakdown products present in analytical extracts and digestion products of pre-processed broccoli (*Brassica olreacea* var. *itálica*) stalks.

Representative UHPLC-ESI-QqQ-MS/MS overlay chromatogram of the analytes monitored at the MRM quantification transitions

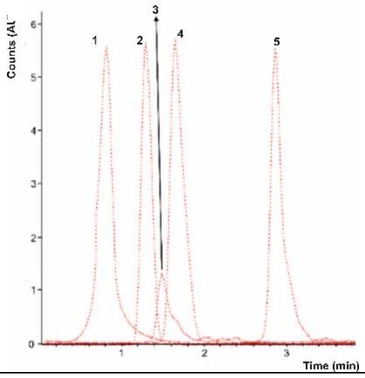

Peak nº	Compound	Retention time (min)	MRM quantitative transition	MRM quantitative transition
1	Erucin	0.820	141.0 > 59.0	161.0 > 70.0
2	Iberin	1.319	164.0 > 105.0	N.d.
3	Indole-3-Carbinol	1.500	130.0 > 77.0	247.1 > 130.1
4	Sulforaphane	1.562	178.0 > 114.0	178 > 71.0
5	3,4-diindolylmethane	2.922	130.0 > 77.0	247.1 > 130.1

ESI, electrospray ionization; MRM, multiple reaction monitoring; N.d., not determined.
